# Electronic control of coherence in a two-dimensional array of photonic crystal surface emitting lasers

**DOI:** 10.1038/srep13203

**Published:** 2015-08-20

**Authors:** R. J. E. Taylor, D. T. D. Childs, P. Ivanov, B. J. Stevens, N. Babazadeh, A. J. Crombie, G. Ternent, S. Thoms, H. Zhou, R. A. Hogg

**Affiliations:** 1 Department of Electronic & Electrical Engineering, Centre for Nanoscience & Technology, North Campus, The University of Sheffield, Broad Lane, Sheffield, S3 7HQ, United Kingdom; 2 James Watt Nanofabrication Centre, James Watt Building, University of Glasgow, University Avenue, Glasgow G12 8QQ.

## Abstract

We demonstrate a semiconductor PCSEL array that uniquely combines an in-plane waveguide structure with nano-scale patterned PCSEL elements. This novel geometry allows two-dimensional electronically controllable coherent coupling of remote vertically emitting lasers. Mutual coherence of the PCSEL elements is verified through the demonstration of a two-dimensional Young’s Slits experiment. In addition to allowing the all-electronic control of the interference pattern, this type of device offers new routes to power and brightness scaling in semiconductor lasers, and opportunities for all-electronic beam steering.

The photonic crystal surface emitting laser (PCSEL) is emerging as a new class of semiconductor light emitter^1^. By contrast to other types semiconductor lasers, the PCSEL utilises the band-edge effect of a two dimensional photonic crystal (PC)^1^,^2^. Where the group velocity of light becomes zero, the light-waves propagating in various directions are coupled with one another, and a 2D standing wave may be established over a broad area. This multi-directional diffraction controls the longitudinal-transverse mode. Semiconductor PCSELs are attracting a great deal of attention, because they have demonstrated large-area coherent lasing^1^,^3^ resulting in a narrow (often diffraction limited), single-lobed beam. PCSELs are also attractive as versatile, single-chip light sources with the capability to control the polarization^2^, beam pattern^4^ and beam direction^5^, and to generate vector beams^6^. Most recently, watt-level output powers have been realized from single emitters^7^.

The first reported PCSEL was based upon organic semiconductor materials^8^, enabling strong reductions in oscillation threshold power and enhancements to slope efficiency for optically pumped organic semiconductor PCSELs^9^. The designs and technologies for PCSELs have been successfully applied to a wide range of inorganic semiconductor materials^10^,^11^,^12^ and emission wavelengths spanning the near-UV^11^,^12^ to THz^13^,^14^, highlighting the importance of this new device type for all laser materials systems. The square lattice photonic crystal is of specific interest here because it can couple light between the two orthogonal in-plane directions and importantly, also couple light to a surface emitting output. Because of these properties, the PCSEL device structure offers the unique opportunity to combine coherent, area scalable, diffraction limited single-mode surface emission with 2D planar photonic circuits. Furthermore, the flexibility afforded by the detailed design of the photonic crystal pattern enables the absolute and relative coupling coefficients of in- and out-of-plane scattering to be controlled.

Over the past 50 years, many methods have been employed to boost the single-mode output power from a diode laser. Although the single mode power that is demonstrated from individual semiconductor lasers keeps rising, these increases have been incremental. Internal optical loss, carrier-photon quantum defect, heat removal, and catastrophic optical damage all impose ultimate limitations. In order to circumvent the latter two of these limitations, arrays of lasers can be used, where the fill factor is reduced to improve thermal management and simultaneously, the active and emitter area is scaled without exceeding the catastrophic optical damage threshold. Most high power pump lasers today are based upon 2D laser arrays in the form of stacks of 1D semiconductor bar arrays with the emission from each element coupled into a large core optical fibre through spatial, wavelength or polarisation methods. These schemes clearly have serious drawbacks in terms of cost and brightness.

To improve brightness, coherence must be maintained between the individual emitters in the array. Coherence coupling methods utilising external optics have been relatively successful for several 10’s of emitters but are not ideal since they greatly increase the system cost and complexity. For lowest cost and large scalability, it is essential that coherence control must be maintained on-chip. In the 1D case, a slab coupled optical waveguide laser (SCOWL) design employs coupling of monolithic emitters to a common slab mode^15^, but this cannot be scaled vertically across bars in a stack. Various in-plane coupling methods have been discussed for monolithic 2D arrays of surface emitters. For example, vertical cavity surface emitting lasers (VCSELs) can be evanescently coupled in plane^16^ but internal loss acts to cause phase jumps between neighbours and control of the fill factor is extremely limited due to the short range nature of evanescent coupling.

A possible candidate for forming large-scale 2D coherent arrays came in the form of grating surface emitting (GSE) laser arrays. Here an array is made of 1D second order Bragg grating coupled lasers^17^. Diffraction limited far-fields may be realized, but there are significant difficulties in scaling to 2D. Multiple Y-branch gain elements^18^, deep etched semiconductor “corner” mirrors^19^, and 2D coupling gratings have been demonstrated^20^ amongst others. However, self-heating effects, making high powers problematic without substrate emission, astigmatism, and the complexity of coupling the elements of the array in 2D are key limiting factors to practical application of this technology.

The PCSEL array described here differs from the GSE array in that the light scattering element now incorporates the gain element. Figure 1 shows a schematic of our device. Here, the PCSEL elements are optically coupled through connecting contacted waveguide regions. This contrasts the GSE that has vertically scattering gratings connecting single-mode wave-guided gain elements. The necessity for single-mode waveguide gain elements in the GSE designs will ultimately limit gain volume and therefore output power. In the device presented here, we are able to harness many of the beneficial properties of PCSELs^1^,^2^,^3^,^4^,^5^,^6^,^7^, in addition to the fabrication technology to achieve optical coupling within the 2D array now being comparatively simple to implement. Furthermore, phase matching conditions between the PCSEL elements may be easily achieved for all elements of the array by designing the coupling waveguides to be of a suitable length. By using such large PCSEL element separation (large as compared to the substrate thickness), thermal isolation of individual elements is achieved. As a consequence, kilowatt class coherent semiconductor laser arrays should be possible utilising PCSEL technology with the added advantage that they emit a coherent beam that can be focused down to the diffraction limit. This significant difference offers the prospect of an increase in brightness by several orders of magnitude over the state-of-the-art. We demonstrate the formation of electronically controlled coherent 2D arrays here through the demonstration of a 2D Young’s Slits experiment.

Our coherently coupled PCSEL array is based upon all-semiconductor epitaxially re-grown PCSELs^21^,^22^. It consists of four PCSEL elements and four coupler regions, all of which are electronically isolated. The four elements of the array are essentially identical in terms of threshold current (65 ± 2 mA), lasing wavelength (991.20 ± 0.06 nm), far-field (<1°), and lasing linewidth (0.50 ± 0.05 nm) (See supplementary information S1).

In this device, coupler regions have exactly the same epitaxial structure as the PCSEL emitters, but the InGaP layer is not patterned with the periodic holes that make up the photonic crystal. The electronic control of these coupler sections is expected to determine the optical isolation between the PCSEL elements, and this control of optical communication between PCSEL elements allows the control of coherence between elements of the array. Measurements on broad area lasers (100 μm width) similar to these couplers indicate an effective transparency current density (where the internal loss is included in the calculation) of 210 ± 10 Acm^−2^ (See supplementary information S2). The effective transparency current density indicates the current density required for light to be coupled between PCSELs. This is a considerably lower current density than that of the lasing PCSEL element, which enables the thermal isolation of the high current density PCSEL elements. For the coupled arrays, two current levels are determined experimentally. The coupler current at which we observed evidence for coherent coupling and injection locking, we refer to as the coupler being in transparency (220 Acm^−2^). Secondly, after a small reduction of this current to the coupler to 200 Acm^−2^ these effects could not be observed, and for this drive condition we refer to the coupler being in loss.

The application of a small change in coupler current therefore allows the switching of communication between PCSEL elements of the array. In addition to coupling, phase matching between the PCSELs must also be achieved to enable coherent outputs. This is shown schematically in Fig. 2 which illustrates the Fabry-Perot mode spacing (indicated by the vertical green lines) of a short and long coupler cavity with regard to the lasing linewidths of two PCSELS of the array (indicated by the blue and red curves). The linewidth of the PCSEL elements here is 0.5 nm. So long as the two PCSELS are tuned such that there is a common area within their linewidth, injection locking can occur. The final requirement for coherent emission is that the in-plane locking light is phase matched from one PCSEL to the other. In order to ensure that the phase matching condition is always met regardless of the refractive index and length of the coupler (both of which will change due to free carrier and thermal effects as coupler current is changed) a coupler length of 1 mm is selected. The Fabry-Pérot mode spacing (from waveguide modeling to deduce the effective index of the mode) for the 1 mm long coupler is calculated as 0.14 nm, resulting in a number of Fabry-Pérot modes within the linewidth of the PCSEL emission (see supplementary S3). As such, one of these coupler modes will always be coincident with the grey area under the lower curve. This large distance between PCSEL elements of the array also guarantees that the PCSELs are thermally isolated. We note that for future use in beam steering applications, much shorter coupler sections (along with technologies to realize transparent coupler sections such as intermixing or re-growth) are required.

Figure 3 shows the near field images of two PCSELs which are projected and overlaid onto a camera in order to explore their mutual coherence. Figure 3(a) shows the case where the two PCSELs are operated sub-threshold (60 mA) and the coupler is in loss (200 mA). Figure 3(b) shows the combined near-field pattern under the same situation (both sub-threshold) but with the coupler now in transparency (220 mA). In both cases, as expected for spontaneous emission sources, there is no evidence for coherence. An increase in intensity is observed for the case where the coupler is in transparency which is attributed to additional amplified spontaneous emission from the coupler and neighbouring PCSEL.

Figure 3(c) shows the same overlapped near-field image with the PCSELs above threshold (70 mA) and the coupler in loss (200 mA). With the coupler in loss, an increased intensity is observed, as compared to the sub-threshold equivalent (Fig. 3(a)), but there is no evidence for mutual coherence. Figure 3(d) shows the same image, but in this case, with the coupler in transparency (220 mA). The small increase in the coupler current to put the coupler into transparency results in the appearance of fringes, separated by 36 ± 4 um. This fringe separation is in excellent agreement with that expected for the dimensions employed in this version of the Young’s slit experiment, 36.5 ± 5 um (see methods and supplementary information S4). This observation not only demonstrates mutual coherence of the two emitters, but additionally confirms coherence over their emitting area for individual PCSELS.

Figure 3(e,f) shows a similar experiment to that reported in Fig. 3(c,d), but now with two PCSELs coupled through a common neighbour and orthogonal coupler elements. In this case the coupling “corner” PCSEL is biased just below its lasing threshold (to ensure transparency) and both couplers are biased to be in loss (Fig. 3(e)) or in transparency (Fig. 3(f)). Similar to the linear coupling regime discussed previously, when the couplers are in loss, there is no evidence for mutual coherence. When the couplers are in transparency, clear diagonal fringes with a separation of 26 um are observed, in excellent agreement with expectation, i.e. 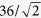 um = 25.4 ± 5 um. This confirms that the couplers can enable coherence between neighbouring PCSELs and also that the PCSEL elements can scatter light coherently round 90° corners, essential for realising a 2D coherently coupled laser array.

In summary, we have reported the realization of a coherently coupled two-dimensional PCSEL array. This is confirmed through the demonstration of mutual coherence, and the electronic control of the form of the interference pattern, verified by a 2 dimensional Young’s Slits experiment. This opens the way for large-scale coherent arrays for ultra high brightness semiconductor sources, and for all electronic two-dimensional beam steering.

## Methods

### Epitxay

This device is fabricated through a two stage epitaxial growth process. Initially the lower n-doped AlGaAs cladding layer (1.5 μm), undoped GaAs/InGaAs triple quantum well active core (containing 38 nm InGaAs quantum wells with 20 nm GaAs barriers), and a p-doped InGaP grating layer (150 nm) are deposited by MOVPE. In regions where a PCSEL is to be realized, a photonic crystal pattern of 150 × 150 μm is transferred to a hard SiO_2_ mask and subsequently the InGaP layer through standard e-beam lithography and dry etching processes. In this case the photonic crystal consists of a circular hole repeated on a lattice with square symmetry. The lattice period is 295 nm and the hole is 240 nm in diameter. The hard mask is removed, and the sample cleaned using HF before being returned to the MOVPE reactor. Here, epitaxial regrowth is used to fill in the etched PC holes with p-doped GaAs, planarise the surface, deposit the upper p-doped AlGaAs cladding layer (1.5 μm), and finally the uppermost p^+^GaAs contact layer (200 nm) of the structure. Further details of the epitaxial process may be found in the supplementary information S3.

### Device Fabrication

Following this second epitaxial step, annular metal contacts (100 μm diameter) are deposited above the PC regions by vacuum evaporation. At the same time, isolated contacts (1 mm long and 100 μm wide) are formed between the PCSEL elements of the array, termed “couplers”. These are separated by 10 μm from the PCSEL contacts via lift-off. Both the PCSELS and couplers are electronically isolated by etching the uppermost p+ GaAs contact layer not protected by the gold ohmic contact. Bond pads are deposited to each separate section in order to provide large electrical contacts for probing. Finally, a common substrate side electrical contact is deposited to provide the n contact to the devices.

### Coherence Measurement

The near field image of a PCSEL array is initially cast through a lens (with 5× magnification in this case) onto a CMOS camera. A mirror is then used to reflect the near field image of one of the devices so that it overlays the nearfield image of the other device. The fringe spacing, of mutually coherent sources is determined by the path length difference, or relative angle, of the two sources. The fringe spacing will be λ/sin(θ) where λ is the wavelength of light in air, and θ is the angle between the two beams at the image plane.

## Additional Information

**How to cite this article**: Taylor, R. J. E. *et al.* Electronic control of coherence in a two-dimensional array of photonic crystal surface emitting lasers. *Sci. Rep.* **5**, 13203; doi: 10.1038/srep13203 (2015).

## Supplementary Material

Supplementary Information

## Figures and Tables

**Figure 1:**
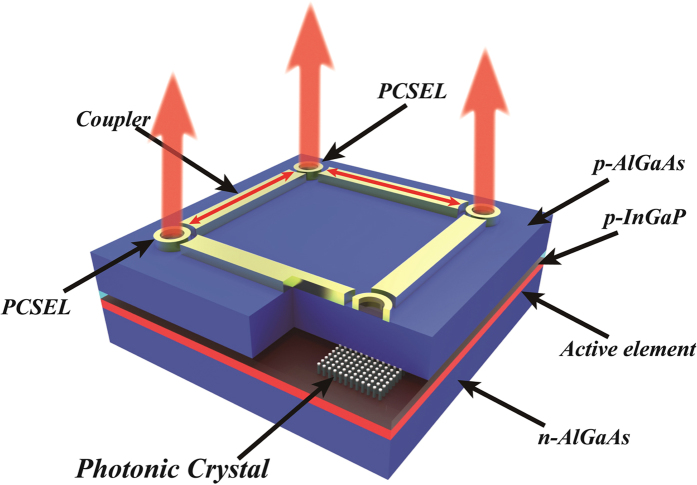
Schematic of the four element PCSEL array. The cut-away is to the level of the first epitaxial step, and shows the position of the photonic crystal lattice which constitutes the PCSEL. Vertical emission from three devices is shown, with lateral coupling via the coupler sections. Two-dimensional coupling of PCSEL elements is achieved through orthogonal scattering at a neighbouring PCSEL.

**Figure 2:**
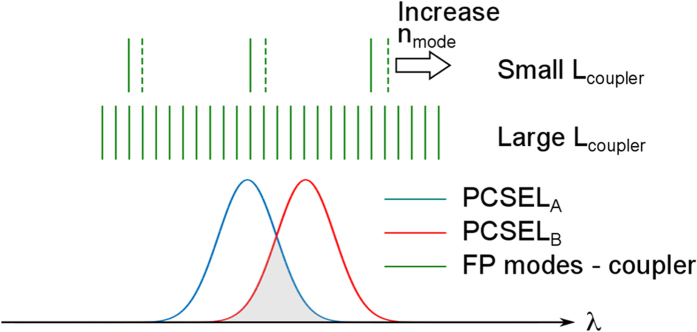
Schematic of the coupler Fabry-Pérot modes (green lines), and lasing linewidths of neighbouring PCSELs (blue and red curves). For sufficiently long coupler lengths, the phase-matching condition between neighbouring PCSELs is met for several Fabry-Pérot modes within the lasing line-width.

**Figure 3:**
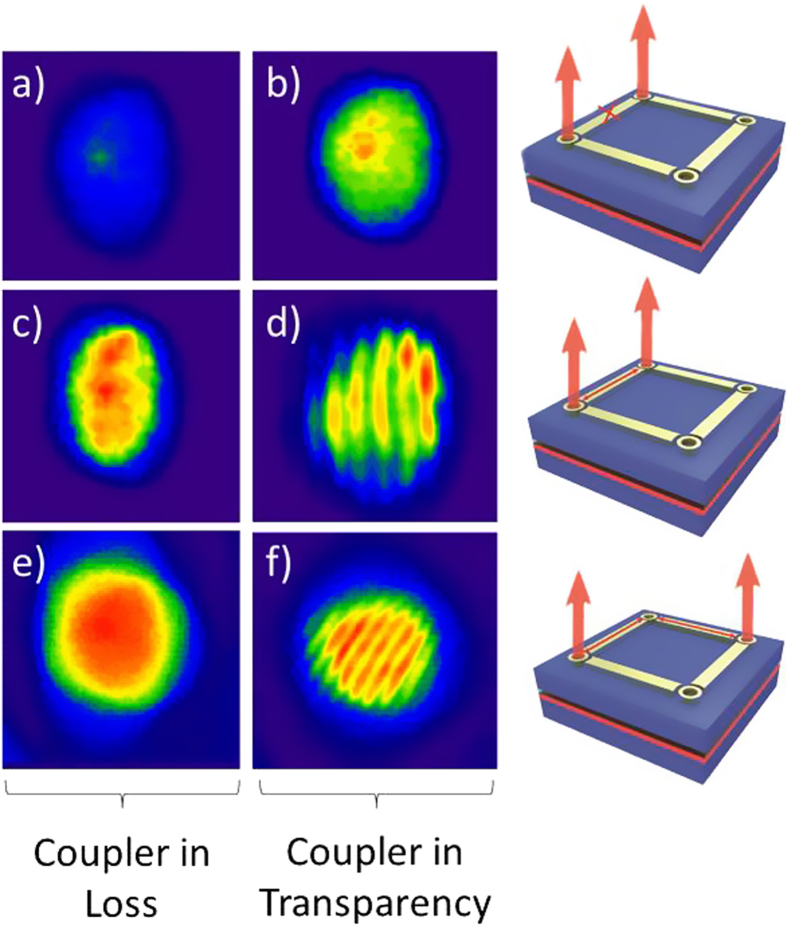
Near field images of two PCSELs overlaid where; (**a**) the two PCSELs are sub-threshold and the coupler is in loss, (**b**) the two PCSELs are subthreshold and the coupler is in transparency, (**c**) the two PCSELs are lasing and the coupler is in loss, (**d**) the two PCSELs lasing and the coupler is in transparency, (**e**) two diagonally separated PCSELs are lasing and the coupler is in loss, (**f**) two diagonally separated PCSELs are lasing and the coupler is in transparency. The right hand column shows a schematic of the drive conditions of the array.
