# Supplementary material for: Electronic control of coherence in a two-dimensional array of photonic crystal surface emitting lasers
Source: Sci Rep. 2015 Aug 20;5:13203. doi: 10.1038/srep13203 (PMC4542471; doi:10.1038/srep13203)
Supplement: Supplementary Information [file srep13203-s1.pdf]

Supplementary Information

Electronic control of coherence in a two-dimensional array of photonic crystal surface emitting lasers

R. J. E. Taylor[1], D.T.D. Childs[1], P. Ivanov[1], B.J. Stevens[1], N. Babazadeh[1], A.J. Crombie[1], G.Ternent[2], S. Thoms[2], H. Zhou[2], R.A. Hogg[1]

[1]Department of Electronic & Electrical Engineering, Centre for Nanoscience & Technology, North Campus, The University of Sheffield, Broad Lane, Sheffield, S3 7HQ, United Kingdom,
[2] James Watt Nanofabrication Centre, James Watt Building, University of Glasgow, University Avenue, Glasgow G12 8QQ

1. PCSEL Element Characterisation

The coherent PCSEL array, as described in the main article, requires all PCSEL elements within the array to have similar operating characteristics. In particular the difference in lasing wavelegnth is required to be less than the lasing linewidth. Here we present charaterisation of individual PCSEL elements, we demonstrate that the four elements from in the 2x2 array have similar light-current (L-I) characteristics, that the difference in lasing peaks is less than the lasing linewidth, and that an individual element has low divergence angle, as expected from the geometry of the PCSEL.

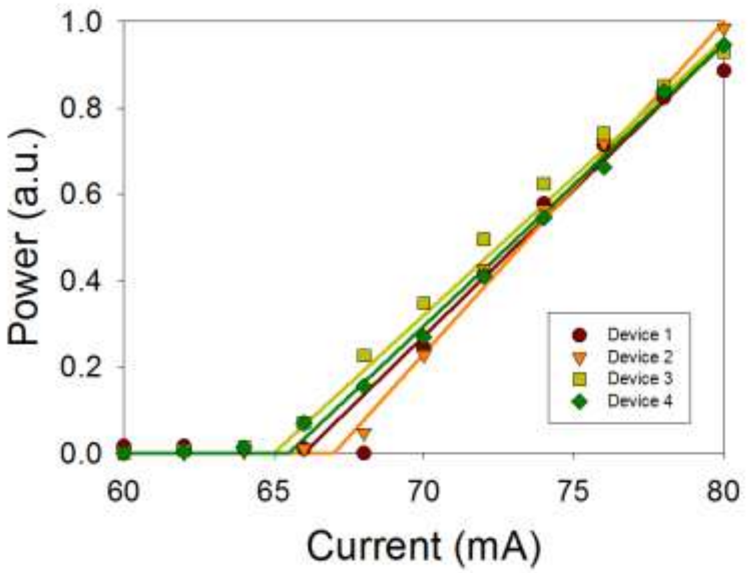

Figure S1.1 L-I curves for all four PCSELs from the same 2x2 array

Figure S1.1 shows the light-current (L-I) characteristics of 4 PCSEL elements from the same 2x2 array operated CW at room temperature. The threshold currents of the 4 PCSEL elements are 65 ±2 mA. Figure S1.2 shows the electro-luminescence (EL) spectra of the same 4 PCSEL elements. Again, the devices are all operated CW at room temperature. The lasing wavelengths are 991.20 ± 0.06 nm, and lasing linewidths are 0.50 ± 0.05 nm. These results demonstrate that within the 2x2 array

the 4 devices are essentially identical with the difference in the lasing wavelength of each PCSEL being significantly smaller than their lasing linewidths.

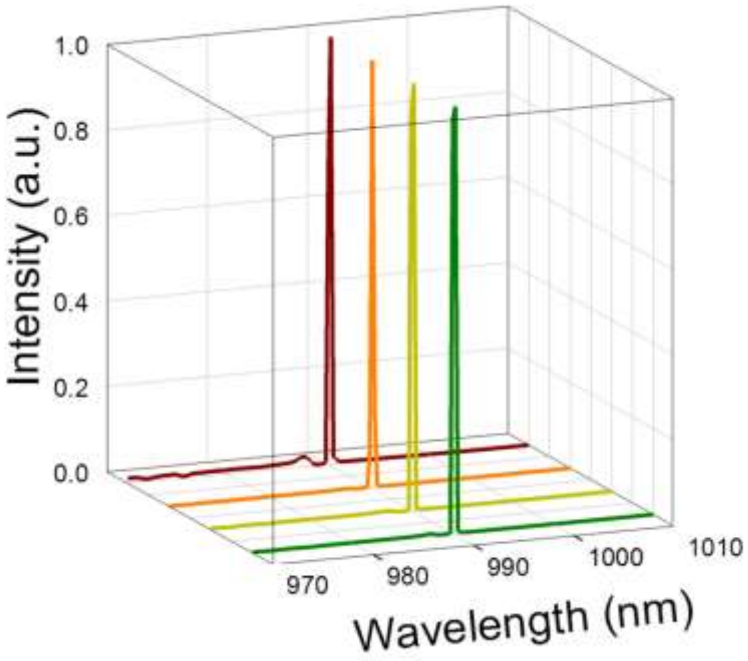

Figure S1.2 Spectra from the four PCSELs from the same 2x2 array at 100mA (~1.5Jth).

Figure S1.3 plots the far-field profile of a single PCSEL operating at 100mA. The FWHM of the emitted beam is ~1.3^{o} in both orthogonal directions, in excellent agreement with diffraction of 1μm wavelength light through a 52 μm diameter aperture.Whilst an anular beam shape is expected, this is not resloved in our measurement. Improved manufacturing methods can be expected to allow this beam shape to be realised.

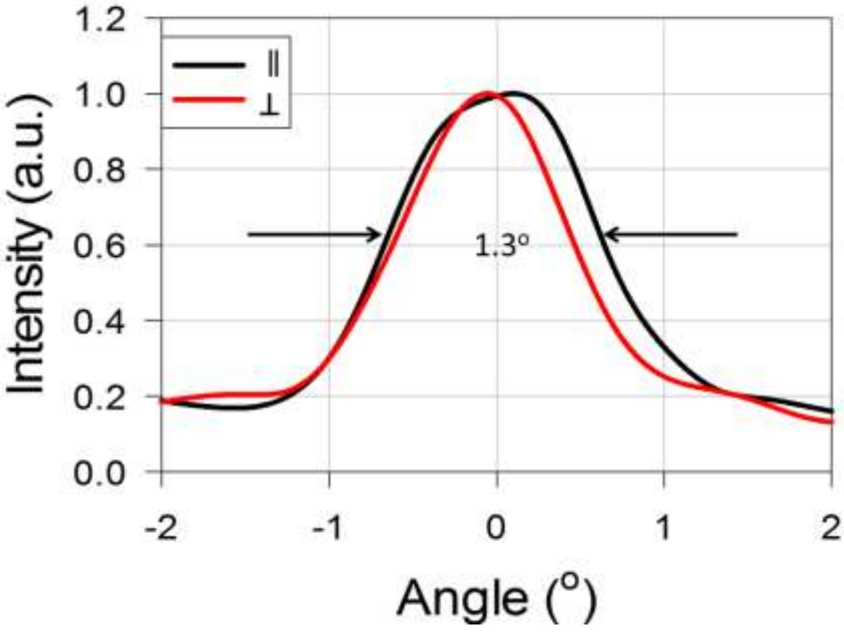

Figure S1.3 Farfield emission profile of an individual PCSEL within the array.

2. Waveguide Modelling & Coupler Characterisation

Figure S2.1 shows a schematic diagram of different parts of the device, showing vertical cross sections of (a) the un-patterned coupler region, and (b) the PCSEL region. The fundamental mode supported by the waveguide is calculated using a fully vectorial mode solver (FIMMWAVE). The normalised mode intensity is superimposed upon the schematic of the physical structure. The mode overlap with the three quantum well active elements (the confinement factor) is determined to be 0.116 and 0.134 for the unpatterned coupler and PCSEL, respectively. The mode index is 3.14 and 3.33 for the unpatterned coupler and PCSEL, respectively.

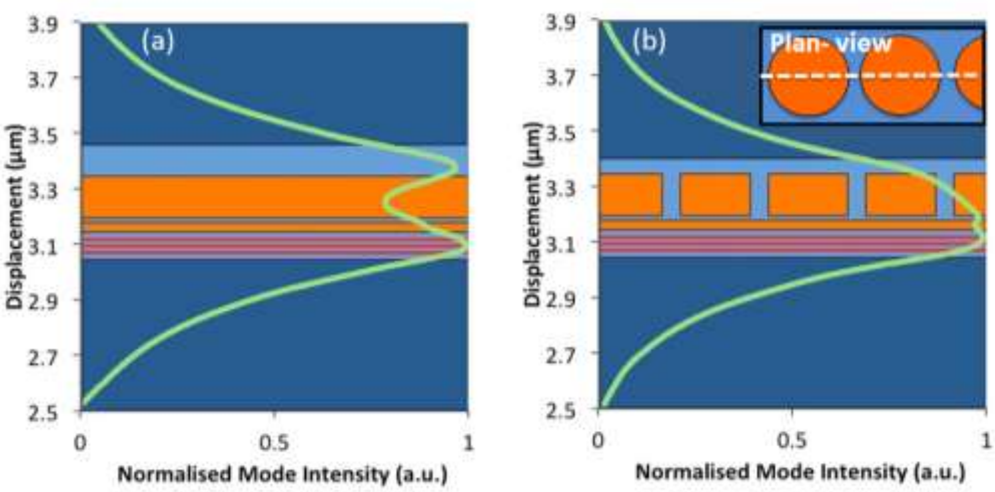

Figure S2.1. Schematic of (a) the unpatterned coupler region, and (b) the PCSEL region. The fundamental mode supported by the waveguide is overlaid on the structure in green. The inset shows the plan view of the vertical cuts represented in (a) & (b).

Figure S2.2 shows a plot of threshold current density of broad area lasers, which have the same structure as the coupler regions, as a function of inverse cavity length. The intercept provides a measure of the effective transparency current density for the couplers, indicating the current density necessary to allow light to be coupled between PCSELs. This transparency point is measured as 220 ±4 Acm^{-2}.

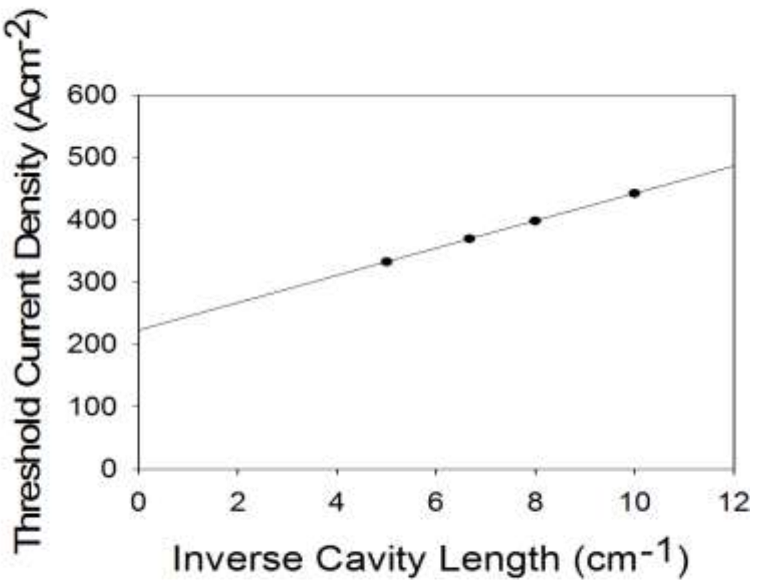

Figure S2.2. Threshold current density as a function of inverse cavity length for edge emitting lasers with the same structure as the coupler region.

3. Coupler Length

Figure S3.1 shows calculated Fabry-Pérot (FP) mode spacing for coupler lengths up to 2mm assuming a mode index of 3.33. As coupler length increases the FP mode spacing decreases. In the present case the FP spacing is 0.14nm which is much less than the lasing linewidth (0.5nm). The device was designed such that the FP mode spacing was significantly less than the line width, ensuring that a phased matched condition is always met within the laser linewidth. If the FP mode spacing is not significantly less than the linewidth then a phase matched condition may not exist between the PCSELs, and can be expected for coupler lengths <300μm.

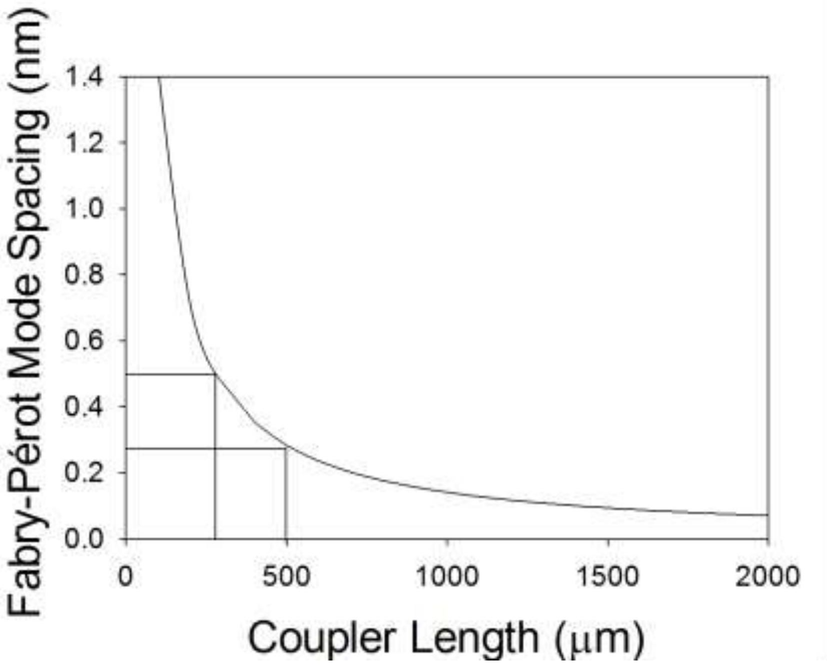

Figure S3.1 Calculated Fabry-Pérot mode spacing as a function of coupler length

4. Epitaxial Processes

Devices considered in this letter have a structure similar to that presented in Wiliams *et al.*[S1], where the photonic crystal region consists of InGaP/GaAs and is realised through epitaxial overgrowth.

The epitaxy was carried out in a Thomas Swan 6x2" close coupled shower head metal organic vapour phase epitaxy (MOVPE) reactor. The reactor is equipped with a Laytec EpiTT emissivity corrected pyrometer, which is crucial in obtaining the temperature control required to grow in a very narrow temperature window. High temperatures are required for native oxide removal, whilst low temperatures are required to minimize arsenic: phosphorous exchange. The EpiTT system is calibrated using a Laytec Absolut, the thermal clean temperature for the regrown wafer is 634°C and the subsequent GaAs/AlGaAs growth must not be allowed to fall below 608°C in order to avoid defects associated with poor planarization of the growth front.

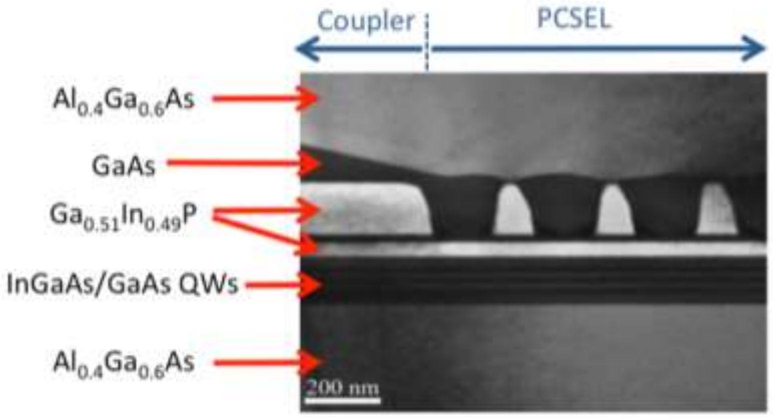

Figure S4.1. Dark-field 002 TEM image (10,000 mag.) at the PCSEL/coupler interface with labels indicating the different materials.

Due to these limitations on varying the growth temperature, in order to achieve conformal infill and planarization of the high aspect ratio photonic crystal structure, we control the mobility of gallium by varying the V:III ratio. A higher V:III ratio for the GaAs growth leads to excellent planarization but also void formation with the PC holes whereas a low V:III ratio leads to excellent conformal infill of the PC holes but at the expense of planarization[S2]. Large reductions in the V:III ratio lead to roughening of the epitaxy, and we have observed that the photonic crystal regions are more tolerant to reduced V:III ratios than the planar regions.

Figure S4.1 shows a TEM image of the regrown structure at the interface between the coupler and PCSEL emitter, showing the lower and upper cladding regions, quantum well active core, photonic crystal, and coupler regions.

5. Spectral Characterisation of the Array

Figure 5.1a shows the spectra of PCSEL array where the current in $PCSEL_A$, $I_{A,}$ is varied from 60mA to 80mA, the coupler is in loss (200mA) and $PCSEL_B$ current is 80mA (i.e. $PCSEL_B$ is lasing). As the current in $PCSEL_A$ is increased the lasing peak of that PCSEL is observed. A red shift of the lasing peak is observed and is attributed

to self-heating of $PCSEL_A$. Figure 5.1b shows the spectra of a array where the current in $PCSEL_A$, I_A, is varied from 60mA to 80mA, the coupler is in Gain (220mA) and the $PCSEL_B$ current is 80mA. The peak observed at low current can be attributed to light traversing from $PCSEL_B$ along the coupler region and being scattered out of $PCSEL_A$. (The inset shows a schematic of the experimental setup). No shift in λ is observed and is attributed to $PCSEL_A$ locking to $PCSEL_B$.

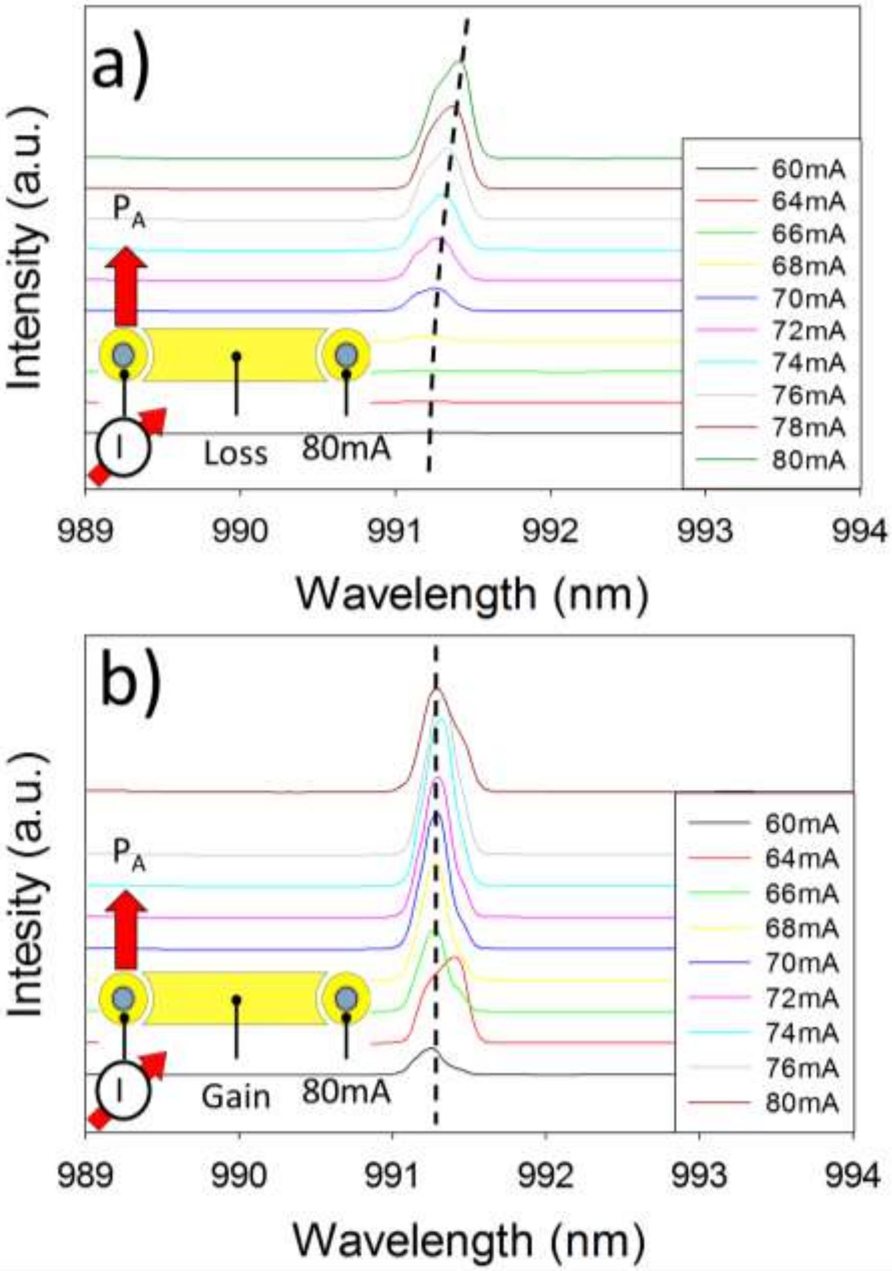

Figure 5.1 Electroluminescence spectra of PCSEL array where the current in $PCSEL_A$ is varied from 60mA to 80mA, the coupler is in loss (a) or Gain (b) and $PCSEL_B$ current is 80mA.

6. Interference

Figure S6.1 shows a schematic of the experimental setup used to measure (or show the absence of) coherence of adjacent PCSELs. a) Shows the near field image of a PCSEL array magnified onto a camera. B) Shows how the insertion of a mirror reflects the near-field image of one PCSEL so that it overlays the image of the other PCSEL. The fringe spacing, of mutually coherent sources is determined by the path length difference, or relative angle, of the two sources. The fringe spacing will be $\lambda/\sin(\theta)$ where $\boldsymbol{\lambda}$ is the wavelength of light in air, and θ is the angle between the two beams at the image plane.

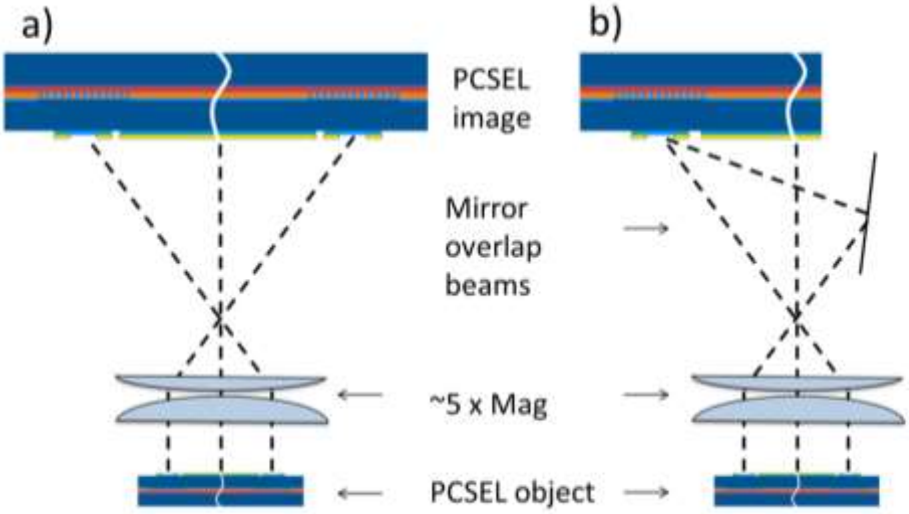

Figure S6.1 Schematic of the setup for coherence control experiment, a) near field image of a PCSEL array magnified onto a camera, b) a mirror reflects the nearfield image of one PCSEL so that it overlays the image of another PCSEL.

References

S1. Williams, D.M. *et al*. Epitaxially regrown GaAs-based photonics crystal surface emitting laser. IEEE Photon. Technol. Lett. **24,** 11, 966-968 (2012).

S2. Taylor, R.J.E. *et al.* Photonic crystal surface emitting lasers based on epitaxial regrowth. IEEE J. Select. Topics Quantum Electron. **19,** 4, 4900407 (2013).
